# Inhibitory Effect of Bisdemethoxycurcumin on DNCB-Induced Atopic Dermatitis in Mice

**DOI:** 10.3390/molecules28010293

**Published:** 2022-12-30

**Authors:** Yanjie Wang, Ping Zhang, Jingyu Zhang, Tie Hong

**Affiliations:** Department of Pharmacology, School of Pharmaceutical Sciences, Jilin University, Changchun 130021, China

**Keywords:** atopic dermatitis, bisdemethoxycurcumin, HaCaT cells, inflammation, MAPK, NF-κB

## Abstract

Atopic dermatitis (AD) is a common chronic inflammatory skin disease. Bisdemethoxycurcumin (BDMC) is an ingredient from the rhizome of the traditional Chinese herbal medicine turmeric. BDMC has been reported to have important pharmacological properties, such as anti-inflammatory, antioxidant, antitumor and antiproliferative activities. However, its effect on atopic dermatitis has not been reported. The purpose of our study was to demonstrate the effectiveness of BDMC on TNF-α/IFNγ-stimulated HaCaT cells and on 2,4-dinitrochlorobenzene (DNCB)-induced AD mice. Our studies showed in vitro that BDMC was able to significantly inhibit the mRNA expression of chemokines and cytokines in TNF-α/IFN-γ-stimulated HaCaT cells and alleviate their inflammatory response. Our studies found in vivo that BDMC was able to significantly improve the symptoms of DNCB-induced AD skin lesions, decrease the number of scratches, ear thickness, and spleen index, improve inflammatory cells and mast cell infiltration and decrease skin thickness. Moreover, it was also able to inhibit the mRNA expression levels of chemokines and inflammatory cytokines and the activation of the MAPK and NF-κB signaling pathways. Thus, the results indicated that BDMC can improve atopic dermatitis in mice and that further clinical studies are warranted on its treatment of AD.

## 1. Introduction

Atopic dermatitis (AD) is a common chronic inflammatory skin disease with complex pathophysiological and clinical heterogeneity in the age of onset, morphology, distribution and severity of lesions [1,2]. The prevalence of AD is about 4% in adults and approximately 10% in children, and 50% of adults with the condition develop persistent skin disease [3]. Recently, many studies have examined the relationships between AD and other conditions, including diabetes, heart disease, hypertension and autoimmune and psychiatric disorders [4,5,6]. These studies have demonstrated that there is a positive relationship between the severity of AD and the prevalence of these disorders [4,5]. The economic burden caused by the chronic recurrence of the disease and family participation in treatment has greatly reduced the living conditions of AD patients and their families.

AD can be caused by an imbalance of regulatory T cells in patients with dermatitis, and the increased differentiation of helper T(Th)2 cytokines such as interleukin (IL)-4 and IL-5, which all inhibit Th1 cell differentiation [7]. Keratinocytes, the main cells of our skin, are stimulated by Th2 cytokines, which may allow immune factors to penetrate damaged skin areas [7,8]. The activation of the MAPK/NF-κB pathway is related to the pathogenesis of allergic reactions [9,10]. Activating the MAPK pathway mainly increases the production of cytokines to stimulate the pro-inflammatory response, and the MAPK pathway regulates the expression of these cytokines by activating the transcription factor NF-κB [11]. Keratinocytes, which are the main epidermal cells, are considered to play a critical role in the pathogenesis of AD. Epidermal keratinocytes function to sustain the recruitment and activation of inflammatory cells, including monocytes, neutrophils, dendritic cells, and T cells, through the production of various inflammatory mediators, such as cytokines and chemokines. The stimulation of keratinocytes with tumor necrosis factor-α (TNF-α) and interferon-γ (IFN-γ) leads to the expression of pro-inflammatory cytokines, chemokines, and adhesion molecules, such as intercellular adhesion molecule-1 (ICAM-1), and these factors contribute to the infiltration of inflammatory cells into sites of inflammation in the skin [12].

Curcumin is the main ingredient extracted from turmeric, a plant known for its medicinal uses. A growing amount of evidence confirms that curcumin might modulate those phenomena involved in the inflammatory, proliferative, and infectious disorders of the skin. In addition to curcumin, bisdemethoxycurcumin (BDMC), another ingredient derived from turmeric, has been proven to exert anti-food allergy and anti-allergic rhinitis effects [13,14,15]. Since BDMC is more stable than curcumin, and has a good water solubility and high permeability, it enters the nucleus more easily to be absorbed by cells [16]. In our previous studies, BDMC was found to inhibit OVA-induced allergic rhinitis (AR) and food allergy (FA) in mice [17,18]. In this study, we examined the effect and mechanism of BDMC on DNCB-induced AD mice and HaCaT cells.

## 2. Results

### 2.1. Effects of Different Concentrations of BDMC Treatment on the Viability of HaCaT Cells

**Figure 1:**
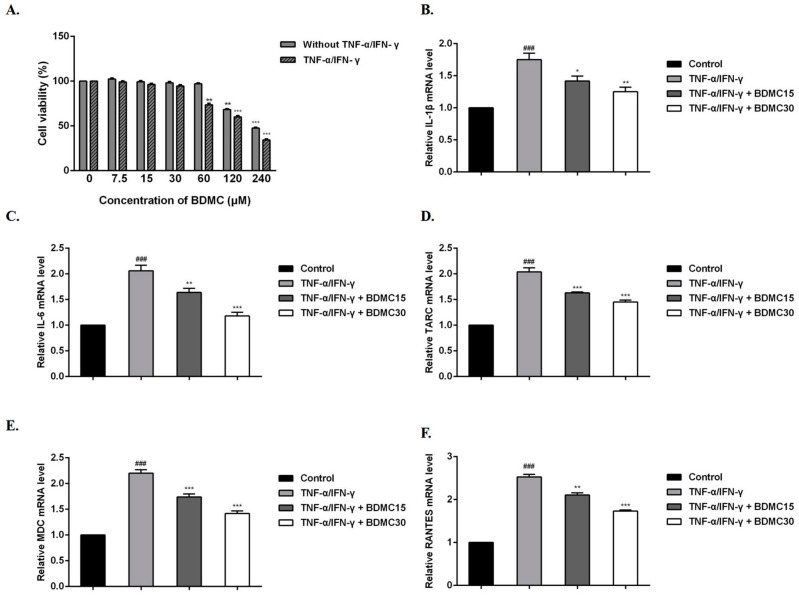
Effects of BDMC on mRNA expression of cytokines and chemokines in HaCaT cells. (**A**) Cells were treated with different concentrations of BDMC (with/without TNF-α/IFN-γ) at 37 °C for 24 h, and then the cell viability was examined by MTT assay. Cells were treated with TNF-α (10 ng/mL)/IFN-γ (10 ng/mL) or equal volume Phosphate Buffered Saline for 24 h; meanwhile, different concentrations of BDMC (15 and 30 µM) were added. Further, the mRNA expression levels of (**B**) IL-1β, (**C**) IL-6, (**D**) TARC, (**E**) MDC, and (**F**) RANTES were determined using RT-qPCR. The RNA gene expression levels of each sample were analyzed three times and normalized to the internal control gene GAPDH. The values are expressed as the means ± standard deviation (SD) of three independent samples. ^###^ *p* < 0.001 vs. control group; * *p* < 0.05, ** *p* < 0.01, *** *p* < 0.001 vs. TNF-α/IFN-γ group.

To examine the effect of BDMC on HaCaT cell viability, an MTT assay was performed. After respective treatments were performed with BDMC (0, 7.5, 15, 30, 60, 120, 240 µM) for 24 h, the results showed that BDMC had no cytotoxicity at the concentration of 60 μM, but showed cytotoxicity in the presence of TNF-α/IFN-γ (Figure 1A). Therefore, we focused on the effects of BDMC at 15 and 30 µM on TNF-α/IFN-γ-induced HaCaT cells in the following experiments.

### 2.2. BDMC Reduces the mRNA Expression Levels of Cytokines and Chemokines in HaCaT Cells

BDMC and TNF-α/IFN-γ were added in vitro to HaCaT cells at the same time for 24 h. RT-qPCR was used to evaluate whether BDMC affected the mRNA expression levels of these cytokines and chemokines after 24 h of treatment with TNF-α/IFN-γ and BDMC. The results showed that the mRNA expression levels of IL-1β, IL-6, TARC, MDC and RANTES were significantly increased in the TNF-α/IFN-γ group compared with the control group. After BDMC treatment, these mRNA expression levels were inhibited and correlated with the dose administered (Figure 1B–F).

### 2.3. BDMC Alleviates the Clinical Symptoms of AD-like Skin Lesions in Mice

**Figure 2:**
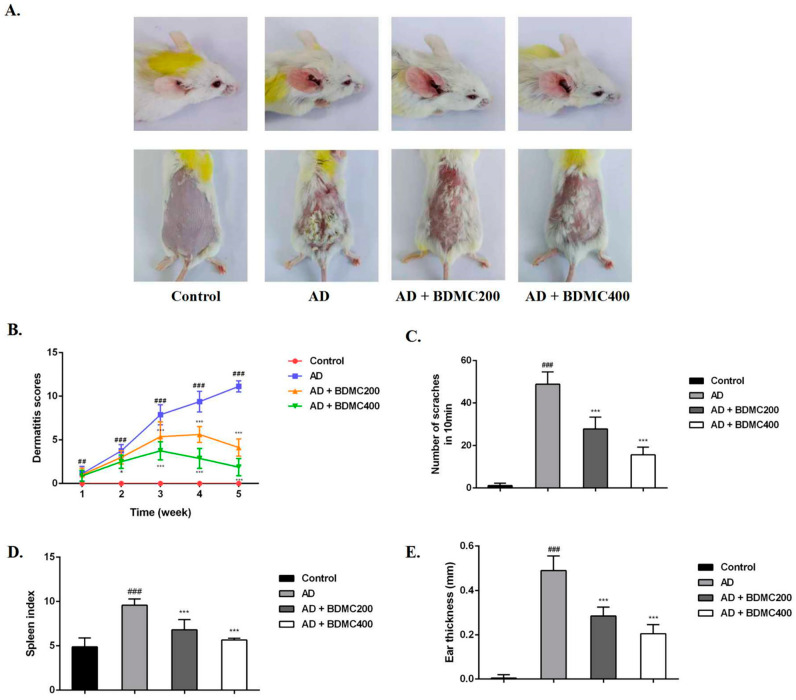
Effects of BDMC on the clinical symptoms of AD-like skin lesions. (**A**) Representative image of AD-like skin and ear lesions on the last day. (**B**) The dermatitis score, including dryness, edema, erythema, and erosion, was calculated once a week. (**C**) The number of scratches was observed for 10 min. (**D**) The spleens of mice were weighed and expressed as spleen index. (**E**) The ear thickness was measured with vernier caliper. The values are expressed as the means ± SD of three independent samples. ^##^ *p* < 0.01, ^###^ *p* < 0.001 vs. control group; * *p* < 0.05, *** *p* < 0.001 vs. AD group.

To investigate the role of BDMC in AD mouse skin lesions, we established DNCB-induced AD models. We found that compared with the control group, the dorsal skin and ears of the mice in the AD group exhibited severe erythema, edema, exfoliation, and scales of skin lesions, and erythema and edema decreased in the mice treated with BDMC (Figure 2A,B). The frequency of scratching behavior and ear thickness in the AD mice were significantly increased compared with the control group, while the frequency of scratching behavior and ear thickness in the BDMC-treated group were significantly reduced compared with the AD group in a dose-dependent manner (Figure 2C,D). Additionally, persistent skin inflammation enlarges the mouse spleen. The spleen index of the AD mice was higher than that of the control group. The daily oral administration of BDMC reduced the spleen index in AD mice in a dose-dependent manner (Figure 2E).

### 2.4. BDMC Decreases the Levels of Serum IgE, IL-4 and IFN-γ

**Figure 3:**
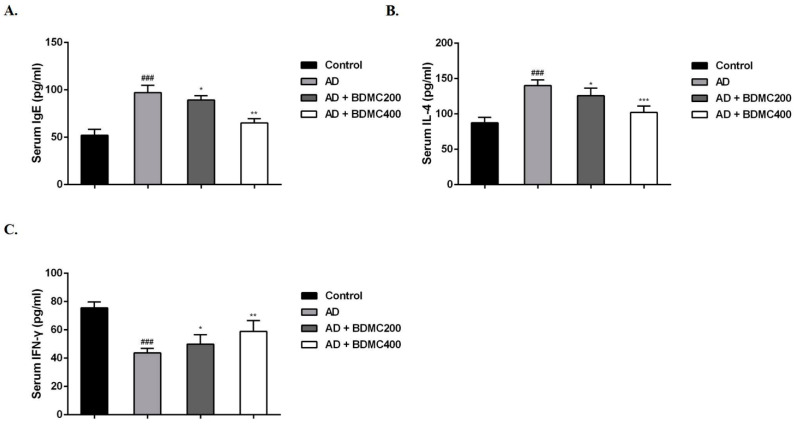
The effect of BDMC on the levels of IgE, IL-4 and IFN-γ in serum. The levels of (**A**) IgE, (**B**) IL-4, and (**C**) IFN-γ in serum were assessed by ELISA. The values are expressed as the means ± SD of three independent samples. ^###^ *p* < 0.001 vs. control group; * *p* < 0.05, ** *p* < 0.01, *** *p* < 0.001 vs. AD group.

The immune response plays a crucial role in the entire pathogenesis of AD. IgE is the main allergic index in the body. To further evaluate the role of BDMC in AD, the expression levels of serum IgE, IL-4 and IFN-γ were measured by ELISA. After stimulation with DNCB, the serum IgE levels of the AD mice were significantly increased compared with those of the control mice. The serum IgE levels of the mice treated with BDMC decreased in a dose-dependent manner (Figure 3A). The serum IL-4 levels of the AD mice were significantly increased, but their IFN-γ levels were significantly decreased compared with those of the control group. Meanwhile, compared with the AD mice, the mice treated with BDMC had significantly reduced serum IL-4 levels and increased serum IFN-γ levels (Figure 3B,C).

### 2.5. BDMC Reduces Skin Hyperplasia and Mast Cell Infiltration in AD-like Skin Lesions

**Figure 4:**
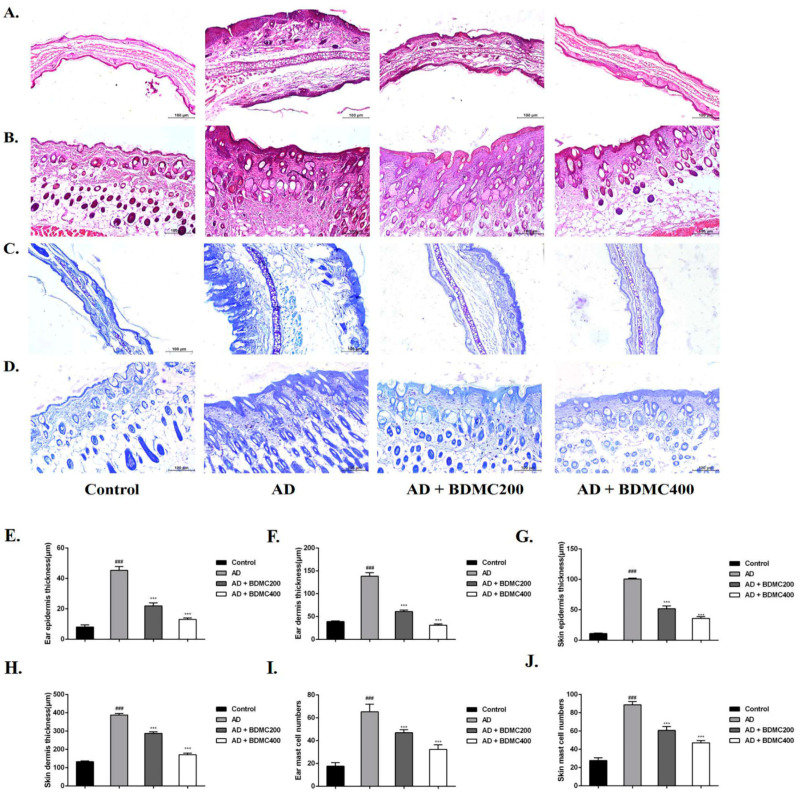
Effects of BDMC on dermal and epidermal thickness and mast cell infiltration were assessed histologically. Histopathological analysis by HE staining (**A**,**B**) and toluidine blue staining (**C**,**D**) of AD-like skin and ear lesions (magnification, ×100; scale bar, 100 µm). (**E**–**H**) The dermal and epidermal thickness was evaluated using HE-stained dorsal and ear tissue microphotographs. (**I**,**J**) Mast cells were counted after toluidine blue staining. The values are expressed as the means ± SD of three independent samples. ^###^ *p* < 0.001 vs. control group; *** *p* < 0.001 vs. AD group.

To assess DNCB-induced histopathological changes, we used hematoxylin and eosin staining (Figure 4A,B). Our results showed that the mice in the control group had thin epidermises, regular dermal collagen, and no inflammatory infiltration. Compared with the control group, the epidermises and dermises of the AD group were significantly thickened, and their dermises exhibited hyperkeratotic and inflammatory cell infiltration. Further, the epidermis and dermis thickness of the mice in the BDMC200 group and BDMC400 group was significantly reduced, and mild dermal inflammatory cell infiltration was observed in these groups (Figure 4E–H). Mast cell infiltration is one of the hallmarks of AD. MCs release multiple cytokines and proinflammatory mediators that exacerbate AD disease progression. Thus, we evaluated the effect of BDMC on mast cells (Figure 4C,D). We found that the number of mast cells in the back skin and ear tissue of the AD mice was significantly increased compared with the control group, and their infiltration was severe. The infiltration of mast cells in the BDMC-treated mice was significantly reduced, and the number of mast cells was also relatively decreased compared with the AD mice (Figure 4I,J). Thus, BDMC treatment reduced epidermal and dermal thickness, as well as DNCB-induced mast cell infiltration.

### 2.6. BDMC Reduces the mRNA Expression of Inflammatory Cytokines in AD-like Skin Lesions

**Figure 5:**
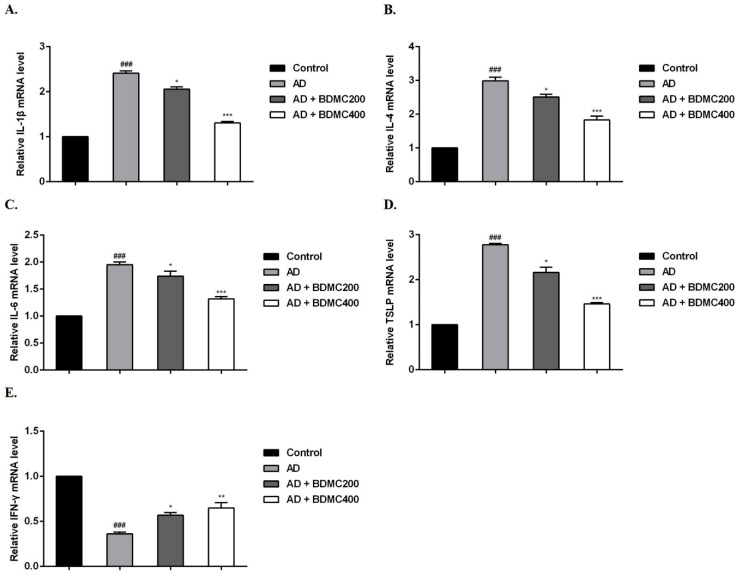
Effects of BDMC on mRNA expression of cytokines in AD-like skin lesions. The mRNA expression of (**A**) IL-1β (**B**) IL-4, (**C**) IL-6, (**D**) TSLP, and (**E**) IFN-γ was determined using RT-qPCR. The RNA gene expression levels of each sample were analyzed three times and normalized to the internal control gene GAPDH. The values are expressed as the means ± SD of three independent samples. ^###^ *p* < 0.001 vs. control group; * *p* < 0.05, ** *p* < 0.01, *** *p* < 0.001 vs. AD group.

Furthermore, we measured the mRNA expression levels of cytokines in mouse dorsal skin lesions. Compared with the control group, DNCB stimulation significantly increased the mRNA expression levels of IL-1β, IL-4, IL-6 and TSLP in the skin lesions of, and decreased the expression levels of IFN-γ in the AD group. Compared with the AD mice, the mRNA expression levels of IL-1β, IL-4, IL-6 and TSLP in the skin lesions of the BDMC-treated mice were decreased, while the expression levels of IFN-γ were significantly increased (Figure 5A–E).

### 2.7. BDMC Inhibits the Activation of MAPK and NF-κB Pathways in AD-like Skin Lesions

**Figure 6:**
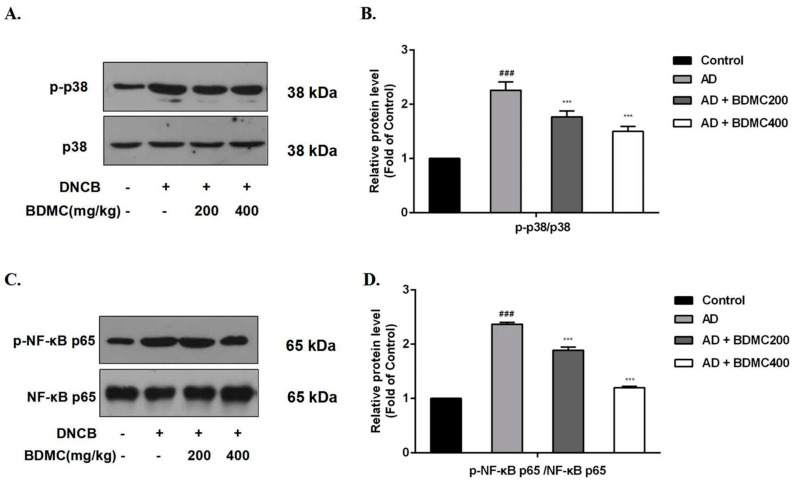
Effects of BDMC on the MAPK and NF-κB pathways. The levels of MAPK pathway-related proteins (**A**,**B**) and NF-κB pathway-related proteins (**C**,**D**) by western blot analysis. The samples were randomly collected from the back skin of three mice in each group, and the density of each band was quantified by ImageJ Software. The values are expressed as the means ± SD of three independent samples. ^###^ *p* < 0.001 vs. control group; *** *p* < 0.001 vs. AD group.

To better evaluate the mechanism of BDMC’s improvement of the skin lesions of the AD mice, the levels of the MAPK-related protein p38 and NF-κB pathway-related protein NF-κB p65 were examined. Our results showed that the phosphorylation level of p38 in the AD group was significantly higher than that in the control group. Further, BDMC prevented the phosphorylation of the p38 protein (Figure 6A,B). However, the effect of BDMC on the JNK and ERK proteins’ phosphorylation remains to be further confirmed. Similarly, the results showed that the phosphorylation level of the NF-κB p65 protein in the AD group was significantly higher than that in the control group. In particular, BDMC prevented the phosphorylation of the NF-κB p65 protein (Figure 6C,D).

## 3. Discussion

AD is a chronic inflammatory skin disease with high recurrence and persistent recurrence rates. Its pathogenesis is mainly related to IgE-mediated hypersensitivity, cell-mediated immune responses, and skin barrier dysfunction [19]. Numerous reports have showed that IFN-γ/TNF-α stimulates epidermal keratinocytes to activate signal transduction pathways, which are involved in promoting inflammation [20,21,22]. HaCaT cellular models stimulated by TNF-α/IFN-γ are widely used to assess potential drug candidates for AD treatment [23]. When the epidermal barrier is damaged, keratinocytes are stimulated, resulting in the expression of many cytokines (IL-1β, IL-33, and TSLP) and chemokines (TARC, MDC) [24,25]. In this study, we explored the therapeutic effect of BDMC on HaCaT cells stimulated with TNF-α/IFN-γ to establish an AD cellular model. The results showed that BDMC was able to significantly inhibit the mRNA expression levels of IL-1β, IL-6, TARC, MDC and RANTES in HaCaT cells stimulated with TNF-α/IFN-γ.

We found that BDMC had ameliorating effects in vivo on dermatitis symptoms in AD mice. An AD mouse model was established that first involved sensitization, followed by DNCB challenge. After repeated stimulation by DNCB, the back and ear skin of the AD mice began to show severe edema, exfoliation, erythema and scaling of the skin lesions, as well as other inflammatory manifestations. The above dermatitis symptoms were significantly improved by BDMC treatment. Itching is one of the common symptoms of AD [26]. We monitored the scratching behavior of the mice in each group within 10 minutes of the last challenge, and found that the AD group had a significant itching response, while BDMC was able to significantly reduce the number of scratches performed by the mice. We also measured the ear thickness of the mice to confirm the effect of BDMC. The spleen is the main immune organ of mice and the center of cellular immunity and humoral immunity. Persistent skin inflammation enlarges the mouse spleen [27]. In this experiment, continuous stimulation with DNCB significantly enlarged the spleens of the mice, and BDMC intervention significantly reduced this spleen enlargement. This result is consistent with our observations of skin performance in mice.

AD is a severe inflammatory skin disease that is accompanied by an increase in the infiltration of inflammatory cells [28]. Skin lesions in AD patients are characterized by the proliferation and infiltration of inflammatory cells, such as T cells, eosinophils, basophils, and mast cells [29]. In the present study, the results of HE staining showed that compared with the control group, the epidermises and dermises of the AD mice were thickened and inflammatory infiltration was increased in this group, while the pathological changes were significantly improved by the oral administration of BDMC in a dose-dependent manner. Mast cell infiltration is one of the hallmarks of AD. MCs release multiple cytokines and proinflammatory mediators that exacerbate AD disease progression [30]. In the present study, the results of toluidine blue staining showed that there was a large amount of MC infiltration in the AD group, and BDMC was able to significantly reduce the occurrence of MC infiltration.

The immune response plays a crucial role in the entire pathogenesis of AD. It is generally believed that an imbalance of Th1/Th2 cells in AD patients leads to Th2 skewing and a subsequent large amount of IgE and IL-4. IgE-mediated hypersensitivity reactions have a large number of inflammatory mediators, such as histamine, which inhibit the production of IFN-γ and further promote the production of IgE. IgE is the main allergic index in the body, and an increase in a patient’s IgE level has become a typical feature of AD. IgE binds to high-affinity receptors on the surface of mast cells [31]. IL-6 and IL-1β are the main proinflammatory cytokines in vivo and participate in innate and adaptive immune responses by regulating the differentiation of immune cells and the production of inflammatory factors [32]. TSLP is an analog of IL-7, which is mainly expressed in human epithelial cells, keratinocytes and bronchial smooth muscle cells. This protein mainly promotes the differentiation of primitive CD4^+^ T cells to Th2 cells through the binding of TSLP receptors on the surface of dendritic cells, and participates in the immune response in vivo [33,34]. TSLP, a crucial factor in AD pathogenesis, activates dendritic cells to promote Th2 immune responses and affects inflammatory cells such as eosinophils and mast cells [35]. In this study, BDMC decreased the levels of IgE and IL-4 and increased IFN-γ levels in DNCB-induced AD mice. The RT-qPCR results showed that BDMC significantly inhibited the mRNA expression levels of IL-1β, IL-4, IL-6 and TSLP in the serum and skin tissue of AD mice, and increased the expression level of IFN-γ in these mice.

The MAPK and NF-κB pathways are involved in various intracellular inflammatory responses [32]. DNCB activates the MAPK signaling pathway and increases the release of proinflammatory cytokines and the activation of some intracellular pathways [36]. NF-κB is a crucial transcription factor that can increase the level of cytokines such as IL-6. NF-κB normally binds to IκB in the cytoplasm and usually exists in an inactive form. IκB is phosphorylated and degraded after activation, and NF-κB enters the nucleus to prepare for gene transcription. The NF-κB pathway is considered a key link in the pathogenesis of AD, which is regulated by the MAPK pathway [37]. Our results showed that BDMC prevented the phosphorylation of p38 proteins in skin lesions; however, its effect on JNK and ERK proteins remains to be further confirmed. Further, our results showed that the effect of BDMC on the MAPK pathway is not simple, and its complex mechanism needs to be further explored. Additionally, the results showed that BDMC prevented the phosphorylation of NF-κB p65 proteins in skin lesions. This study suggests that inhibiting the activation of the MAPK and NF-κB signaling pathways may be a potential mechanism by which BDMC alleviates AD.

In conclusion, our study showed that BDMC has an inhibitory effect on DNCB-induced AD-like skin lesions. The underlying mechanism of this may involve BDMC inhibiting the activation of the MAPK and NF-κB pathways. Additionally, BDMC was found to have a protective effect on TNF-α/IFN-γ-stimulated HaCaT cells. This discovery suggests that BDMC may be of long-term significance in the systematic study and effective treatment of AD.

## 4. Methods

### 4.1. Mice

In this study, we utilized 32 female BALB/c mice (six-week-old) purchased from Liaoning Changsheng Biotechnology Co., Ltd. (Shenyang, China). The mice were maintained under specific pathogen-free conditions in an air-conditioned room, and had free access to food and water. The procedures were approved by the Institutional Animal Care and Use Committee of Jilin University (NO. 20200060).

### 4.2. Cell Culture and Treatment

Human keratinocytes (HaCaT cells), provided by the National Infrastructure of Cell Line Resource, China, were cultured in Dulbecco’s Modified Eagle Medium (DMEM) with 10% heat-inactivated FBS and 1% penicillin-streptomycin at 37 °C in a 5% CO_2_ incubator.

### 4.3. Cell Viability Assay

HaCaT cells (1 × 10^4^ cells/mL) were seeded in a 96-well plate overnight at 37 °C. Subsequently, cells were treated with different concentrations of BDMC (which was purchased from Sigma-Aldrich, St. Louis, MO, USA) with a purity of more than 98% (HPLC) (0, 7.5, 15, 30, 60, 120, 240 μM) and with or without TNF-α/IFN-γ (10 ng/mL) for 24 h. After treatment with BDMC, 100 μL MTT (10 mg/mL) reagent was added to the well, and the cells were incubated at 37 °C for 4 h. Then, we dissolved the crystal violet with DMSO (200 μL/well). Finally, absorbance intensity was quantified at 570 nm using a microplate reader.

### 4.4. Induction of AD Mouse Model

**Figure 7:**
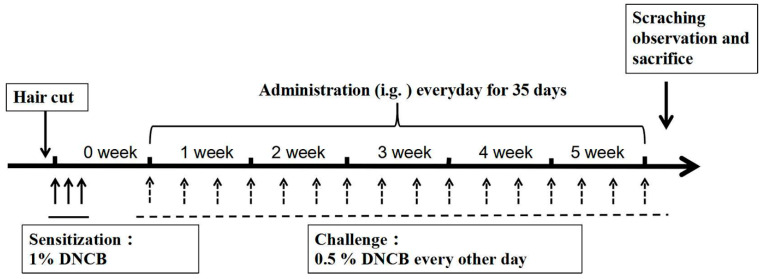
Schematic illustration of AD induction and BDMC treatment. Except for the control group, all mice were sensitized with 200 μL and 30 μL 1% DNCB on their back skin and right ears, respectively, for three consecutive days. After 4 days, the back skin and right ears of each mouse were challenged with 0.5% DNCB every other day for 5 consecutive weeks.

To induce AD-like skin lesions, 32 mice were randomly divided into 4 groups (n = 8) as follows: (1) Control (vehicle treatment), (2) AD (DNCB treatment), (3) AD + BDMC200 (BDMC 200 mg/kg), and (4) AD + BDMC400 (BDMC 200 mg/kg). As previously described [38,39] and depicted in Figure 7, the dorsal skin and right ears of mice were challenged by DNCB. Briefly, we shaved the back hair of the mice in advance. Except for the control group, all mice were sensitized with 200 μL and 30 μL 1% DNCB dissolved in acetone–olive oil solution (3:1) on their back skin and right ears, respectively, for three consecutive days. After 4 days, the back skin and right ears of each mouse were challenged with 0.5% DNCB every other day for 5 consecutive weeks.

### 4.5. Drug Treatment

The mice in BDMC treatment group were orally treated with 200 and 400 mg/kg of BDMC, respectively (dissolved in 1% carboxy methyl cellulose at the concentration of 20 mg/mL and 40 mg/mL, respectively), once a day from the 1st week to the 5th week. The mice in other groups were only treated with 1% carboxy methyl cellulose. All mice were sacrificed by cervical dislocation 24 h after the last challenge.

### 4.6. Atopic Dermatitis Score, Scratches, Ear Thickness and Spleen Index

As previously described, the symptoms of erythema, edema, exfoliation, and scales of skin lesions were scored from 0 to 3 according to severity [40]. Meanwhile, the frequency of scratching behavior in each group was observed for a duration of 10 min. The thickness difference of each mouse’s right ear was measured by using a Vernier caliper 24 h after the last DNCB stimulation, and serum, spleen samples, and dorsal skin were collected.

### 4.7. Histological Analysis

The mouse dorsal skin and right ear tissues were resected and paraffin-embedded. Sections (4 µm thick) were then stained with hematoxylin and eosin (H&E) for histopathological observations. After staining, 5 tissues were randomly selected for epidermis and dermis thickness analysis. Other sections were stained with toluidine blue to determine the number of mast cells. Images were obtained using a microscope (Leica DM2500, Berlin, Germany, magnification, ×100) to evaluate the sections.

### 4.8. ELISA

Serum immunoglobulin (Ig) E and the cytokines IL-4, TSLP and interferon-γ (IFN-γ) were measured using the appropriate ELISA kits (Shanghai Enzyme-linked Biotechnology Co., Ltd., Shanghai, China). The procedures were conducted according to the manufacturer’s guidelines.

### 4.9. Real-Time Polymerase Chain Reaction

Gene expression in dorsal skin was detected by RT-qPCR. Total RNA was extracted from the mouse dorsal tissue using an RNAeasy^TM^ Animal RNA Extraction Kit (Beyotime, Beijing, China) according to the manufacturer’s instructions. According to the operating procedure of the MonScript^TM^ RTIII All-in-One Mix (Monad Biotech Co., Ltd., Suzhou, China), the operation was performed according to the specified conditions to convert RNA into cDNA. Furthermore, the cDNA analysis was performed with MonAmp^TM^ qPCR Mix using the ABI Real-Time PCR system (Applied Biosystems, Foster City, CA, USA). The primer sequences are shown in Table 1. The RNA gene expression levels of each sample were analyzed three times and normalized to the internal control gene GAPDH.

### 4.10. Western Blot Assay

To detect the protein activity, we isolated the total proteins from the mouse dorsal skin tissue with RIPA buffer (Genstar, Beijing, China) supplemented with protease inhibitor PMSF (Genstar, Beijing, China). Protein concentrations were quantified with a BCA Protein Assay kit (Beyotime, Beijing, China). Proteins (20–30 µg) were electrophoresed on 10% SDS-PAGE and then transferred onto the PVDF membranes (Beyotime, Beijing, China), which were then put into TBST containing Tris-buffered saline, 0.1% (*v*/*v*) Tween 20 solution and 5% nonfat dry milk; they were then shaken for 2 h at room temperature. The membranes were then incubated with primary antibodies (1:1000, BIOSS, Beijing, China) overnight at 4 °C. Finally, the membranes were incubated with specific HRP-conjugated secondary antibodies (1:4000, Beyotime, Beijing, China) for 1 h at RT followed by visualization using an enhanced chemiluminescence detection reagent. All the bands on the blots were quantified using ImageJ (version 1.51j8, National Institutes of Health, Bethesda, MD, USA).

### 4.11. Statistical Analysis

The experimental analyses were performed using the SPSS 20.0 statistical software package (IBM Corp.) and data were expressed as the means±standard deviations (SDs). One-way analysis of variance was used to evaluate significant differences between multiple groups. For dermatitis scores, Kruskal–Wallis test followed by Dunn’s multiple comparison test was used. *p* < 0.05 was considered to indicate a statistically significant difference.

## Figures and Tables

**Table 1:** Primer sequences for RT-qPCR.

Genes	Forward	Reverse
GAPDH	CTG CTC CTC CTG TTC GAC AGT	CCG TTG ACT CCG ACC TTC AC
mIL-4	TAC CAG GAG CCA TAT CCA CGG ATG	TGT GGT GTT CTT CGT TGC TGT GAG
mIFN-γ	GAG CCT AGA GAC TAT CAC ACC G	TAC CAG AGG GTG TAG TTA GCG G
mIL-1β	TGG ACC TTC CAG GAT GAG GAC A	GTT CAT CTC GGA GCC TGT AGT G
mIL-6	AGT TGC CTT CTT GGG ACT GA	TCC ACG ATT TCC CAG AGA AC
mTSLP	AGC TTG TCT CCT GAA AAT CGA G	AGG TTT GAT TCA GGC AGA TGT T
hIL-1β	CTC TCA CCT CTC CTA CTC ACT	ATC AGA ATG TGG GAG CGA AT
hIL-6	CGA GCC CAC CGG GAA CGA AA	GGA CCG AAG GCG CTT GTG GAG
hTARC	GTC TTG AAG CCT CCT CAC CC	GGA TCT CCC TCA CTG TGG CT
hMDC	GTT GTC CTC GTC CTC CTT GC	GGA GTC TGA GGT CCA GTA GAA GTG
hRANTES	CGC TGT CAT CCT CAT TGC TA	GCA CTT GCC ACT GGT GTA GA

## Data Availability

Not applicable.
